# Potential of jackfruit inner skin fibre for encapsulation of probiotics on their stability against adverse conditions

**DOI:** 10.1038/s41598-023-38319-y

**Published:** 2023-07-10

**Authors:** Kantiya Petsong, Pensiri Kaewthong, Passakorn Kingwascharapong, Krisana Nilsuwan, Supatra Karnjanapratum, Patcharaporn Tippayawat

**Affiliations:** 1 Department of Food Technology, Faculty of Technology, Khon Kaen University, Khon Kaen, 40002 Thailand; 2 Department of Agro-Industry, School of Agricultural Technology, Food Technology and Innovation Research Centre of Excellence, Walailak University, Thasala, Nakhon Si Thammarat, 80161 Thailand; 3 Department of Fishery Products, Faculty of Fisheries, Kasetsart University, Bangkok, 10900 Thailand; 4 International Center of Excellence in Seafood Science and Innovation, Faculty of Agro-Industry, Prince of Songkla University, Songkhla, 90110 Thailand; 5 Food Innovation and Packaging Center, Chiang Mai University, Chiang Mai, 50100 Thailand; 6 Department of Medical Technology, Faculty of Associated Medical Sciences, Khon Kaen University, Khon Kaen, Thailand

**Keywords:** Biotechnology, Microbiology

## Abstract

The aim of this study was to investigate the impact of jackfruit inner skin fibre (JS) incorporated with whey protein isolate (WPI) and soybean oil (SO) as a wall material for probiotic encapsulation to improve probiotic stability against freeze-drying and gastrointestinal (GI) tract conditions. *Bifidobacterium bifidum* TISTR2129, *Bifidobacterium breve* TISTR2130, and *Lactobacillus acidophilus* TISTR1338 were studied in terms of SCFA production and the antibiotic-resistant profile and in an antagonistic assay to select suitable strains for preparing a probiotic cocktail, which was then encapsulated. The results revealed that *B. breve* and *L. acidophilus* can be used effectively as core materials. JS showed the most influential effect on protecting probiotics from freeze-drying. WPI:SO:JS at a ratio of 3.9:2.4:3.7 was the optimized wall material, which provided an ideal formulation with 83.1 ± 6.1% encapsulation efficiency. This formulation presented > 50% probiotic survival after exposure to gastro-intestinal tract conditions. Up to 77.8 ± 0.1% of the encapsulated probiotics survived after 8 weeks of storage at refrigeration temperature. This study highlights a process and formulation to encapsulate probiotics for use as food supplements that could provide benefits to human health as well as an alternative approach to reduce agricultural waste by increasing the value of jackfruit inner skin.

## Introduction

Encapsulation is an effective technique to protect living microorganisms from harsh conditions^1^. Polysaccharide-based materials such as alginate, pectin, and carrageenan^2–4^ have been reported to be potential wall materials for encapsulating microorganisms. An increase in the value of agricultural waste is an alternative approach to reduce the carbon footprint caused by climate change^5^. Jackfruit (*Artocarpus heterophyllus*) is widely cultivated in several countries in Southeast Asia, including Thailand. Approximately 65–80% of the total weight of jackfruit is waste^6^. It has been reported that the inner skin of jackfruit is a natural fibre containing a large amount of non-digestible polysaccharides and a major source of prebiotics^7^. Moreover, this agricultural waste contains a large amount of cellulose and pectin^8^, which are excellent wall materials for microorganism encapsulation^9, 10^. Based on its functional properties, jackfruit inner skin fibre might protect beneficial microorganisms from the human gastrointestinal (GI) tract as well as provide health benefits. When encapsulating probiotics, a dry powder is the most convenient form with which extend shelf life, as it allows easy storage and transport^11^. However, the transformation of probiotics into a dry powder is the major cause of the loss of living microorganisms^10, 12^, which is the pain point of probiotic materials. Protein- and lipid-based materials have been reported to be effective wall materials to protect microorganisms from drying conditions. Previous evidence^13, 14^ has indicated that a combination of protein, lipid, and natural fibre materials from jackfruit inner skin could be of interest for the development of a potential wall material for probiotic encapsulation.

*Bifidobacterium* and *Lactobacillus* have been reported as the most widely used probiotic bacteria included in many functional foods and dietary supplements^15^ and are associated with a low health risk after human consumption^16^. Several research studies have demonstrated that these probiotics affect the composition of the gut microbiota, which results in a loss of total body weight^17^ because of their ability to produce short-chain fatty acids (SCFAs; includes acetic acid, butyric acid, and propionic acid).

This study aimed to evaluate the potential of jackfruit inner skin fibre (JS) as wall material incorporating with commercial materials (whey protein isolate (WPI) and soybean oil (SO)) on probiotic protection ability against harsh conditions (freeze-drying, gastro-intestinal tract). *B. bifidum* TISTR2129, *B. breve* TISTR2130, and *L. acidophilus* TISTR1338 were screened and selected for preparing a probiotic cocktail as core material based on their ability to produce SCFAs, antibiotic-resistant profiles and their relationship by antagonistic assay. The morphology of encapsulated probiotics was characterized by using a scanning electron microscope (SEM), while the storage stability of encapsulated probiotics was also evaluated at ambient and refrigeration temperatures for 8 weeks.

## Materials and methodology

### Chemicals and reagents

All antibiotics used in the antibiotic sensitivity test and the reagents for GI tract simulation (pepsin, bile salt, and pancreatin) were obtained from Sigma Chemical Co. (St. Louis, MO, USA). WPI (Davisco Foods International, USA), SO (purchased from a supermarket), and JS (purchased from a local market in Khon Kaen, Thailand) were used as wall materials for encapsulation.

### Screening and preparation of the probiotic cocktail for the encapsulation study

#### Bacterial strain and growth conditions

Probiotic strains, including *B. bifidum* TISTR2129, *B. breve* TISTR2130, and *L. acidophilus* TISTR1338 (Table 1), were purchased from the TISTR Culture Collection, Thailand. The probiotic strains were stored as working stocks at − 30 °C. The cultivation of all probiotic strains was described by Chang et al.^18^. *Bifidobacterium* were cultured in De Man, Rogosa and Sharpe (MRS) (HiMedia Laboratories Pvt. Ltd., India) supplemented with 0.05% l-cysteine hydrochloride (Sigma‒Aldrich, St. Louis, Mo, USA). The *Lactobacillus* cultures were grown in MRS medium without supplementation. All cultures were incubated at 37 °C for 36–60 h under anaerobic conditions.

**Table 1:** Bacterial strains used in the study.

Bacterial strain	Source of isolation	Reference
*Bifidobacterium bifidum* TISTR2129	Stool of a breast fed infant	TISTR Culture Collection
*Bifidobacterium breve* TISTR2130	Intestine of an infant	TISTR Culture Collection
*Lactobacillus acidophilus* TISTR1338	Poultry intestine	TISTR Culture Collection

#### Short-chain fatty acid production study

All three tested probiotic strains were cultured as described above for 5 days. Supernatant samples of the probiotics cultured for 1, 3, and 5 days were filtered with a nylon syringe filter with a pore size diameter of 0.45 µm. The method for the determination of acetic acid, butyric acid, and propionic acid followed Chang et al. with modifications^18^. The filtrates (2 µL) were injected into a 7890A series gas chromatography system (Agilent Technologies, Santa Clara, CA, USA) equipped with a DB-FFAP capillary column (30 m × 0.25 mm, 0.25 µm) and a flame ionization detector (FID) at a split ratio of 1:5. The analytical conditions were an injection port temperature of 250 °C and detector temperature of 250 °C. The oven temperature was programmed to increase from 100 to 180 °C at a rate of 10 °C/min (no hold) and from 180 to 250 °C at a rate of 30 °C/min (hold for 3 min). The flow rate of helium gas was 1.5 mL/min. The chromatographic peaks of the samples were identified from the retention times of the standards and computed with Agilent OpenLAB CDS ChemStation GC Drivers A.01.03 software. Each sample was analysed with two replicates.

#### Antibiotic sensitivity test

To study the antibiotic-resistant profiles of all tested probiotic strains, the broth dilution method was performed as described by Petsong et al.^19^ The experiment was conducted with three biological replicates. To prepare antibiotic stock solutions, ampicillin (256 µg/mL), gentamicin (4096 µg/mL), streptomycin (4096 µg/mL), and vancomycin (256 µg/mL) were dissolved in water. Erythromycin (256 g/mL) and tetracycline (1024 µg/mL) were dissolved in absolute ethanol. The sensitivity of the probiotic strains (10^5^ CFU/mL) to each antibiotic was evaluated using 96-well microtiter plates containing twofold serial dilutions of each antibiotic (ampicillin, gentamicin, erythromycin, streptomycin, and tetracycline). Each mixture was incubated at 37 °C for 24 h under anaerobic conditions. The growth of the probiotic strains was observed at 600 nm using a microplate reader (Spectramax-M3, USA).

#### Antagonistic assay

An antagonistic assay was performed using the disk inhibition method following a previous study^20^ with modifications. *B. bifidum*, *B. breve*, and *L. acidophilus* were grown in suitable medium as described above. The inoculum was incubated at 37 °C under anaerobic conditions to obtain approximately 10^8^ CFU/mL. Each potential sensitive strain was streaked on MRS agar plates using a sterile cotton swab. Paper disks (Whatman no. 4; Sigma–Aldrich, 5 mm) containing 20 µL of a possible producer of inhibitory substances was placed on prepared agar plates. The growth of all tested strains was determined after incubation for up to 72 h. The presence of clear zones around the inoculated disks was recorded as an inhibitory phenomenon by the bacteria contained in the disks against bacteria streaked on the agar plates. Paper disks containing incubated medium without any culture (20 µL) were used as the control. The experiment was conducted with three biological replicates. Probiotic strains that showed no negative relationship with the others were selected to study encapsulation.

#### Preparation of a probiotic cocktail

*Bifidobacterium breve* and *L. acidophilus* were selected to develop the core material to be encapsulated, since no antagonism was observed between these strains. Probiotic strains were cultured as described above. Cultures in the log phase were centrifuged, and the cells were washed with 0.85% NaCl three times before use. The ratio of *B. breve* to *L. acidophilus* was 1:1 (approximately 10^9^ CFU/mL), and the mixture was suspended in 0.85% NaCl (10 mL) to prepare a probiotic suspension to study encapsulation.

### Development of a wall material for probiotic encapsulation with enhanced protection provided by jackfruit inner skin fibre

To obtain an effective formulation of encapsulated probiotics that could survive under harsh conditions (freezing and GI tract conditions), this study was designed to evaluate the effect of wall materials (WPI, SO, and JS) on the survivability of the tested probiotics. The experiment followed the procedure of Petsong et al.^21^ with minor modifications. Various ratios of WPI, SO, and JS were used to obtain 10 g of each mixture (for the 13 formulations representing 10 different combinations, see Table 2) designed using Design-Expert version 13 (Stat-Ease Inc., Minneapolis, USA). The optimal formulation was designed based on the positive and negative effects of the individual wall materials and their combinations on the survivability of the probiotics after freeze-drying. JS was prepared by washing with tap water to eliminate the grime before being finely chopped. The moisture in JS was evaporated by using a hot air oven (60 °C, 48–72 h) before blending to obtain JS as a dry powder. All materials used for encapsulation were autoclaved (121 °C, 15 min) to eliminate the initial contaminating microorganisms. To unfold the protein structure, WPI was rehydrated in sterilized distilled water (80 mL) and incubated at 4 °C overnight. The prepared probiotic suspension and SO were added to the prepared WPI (heating to 80 °C for 30 min and cooling at room temperature for 45 min). The desired amount of JS was then added to the mixture. Each mixing step was conducted after 10 min of agitation at 2400 rpm using a homogenizer (Nihon Seiki Kassha, Ltd, Japan). Each mixture was kept at − 60 °C before being tempered at − 50 °C using a laboratory-scale freeze-dryer (Gamma 2-16LSC, Christ, Germany) for 48–72 h. The dry powders of each encapsulated probiotic formulation were prepared in three separate batches.

**Table 2:** Various ratios of whey protein isolate (WPI), soybean oil (SO) and jackfruit inner skin fibre (JS) used to obtain 10 g of a mixture for probiotic encapsulation.

Formulation	WPI:SO:JS (g)
1	6.67:1.67:1.67
2	5:5:0
3	10:0:0
4	10:0:0
5	3.33:3.33:3.33
6	1.67:6.67:1.67
7	0:5:5
8	0:10:0
9	1.67:1.67:6.67
10	0:0:10
11	5:0:5
12	0:0:10
13	0:10:0

### Survivability of encapsulated probiotics after freeze-drying

The method to enumerate the number of encapsulated probiotics after freeze-drying was carried out according to a previous study^10^ with minor modifications. Probiotic powder (1 g) was mixed with 30 mL of 1 M sodium phosphate buffer (pH 7.5) supplemented with 0.75% Tween® 20. The mixture was agitated at 200 rpm for 60 min at room temperature. A spread plate technique was performed on MRS agar, and the samples were incubated at 37 °C under anaerobic conditions for 48–72 h. The survivability of the encapsulated probiotics after freeze-drying (encapsulation efficiency; EE%) was calculated as follows. The number of probiotics recovered from the powder (RP) was divided by the initial number of probiotics added for encapsulation (IP) and then multiplied by 100:

$$EE \left( \% \right) = \frac{RP}{{IP}} \times 100$$

### Characterization and stability studies of the encapsulated probiotics

#### Morphology and surface characterization of the encapsulated probiotics

Morphology and surface characterization of the encapsulated probiotics (including the optimal formulation, WPI-SO formulation, and JS-SO formulation) were examined using field-emission scanning electron microscopy (FE-SEM) (TESCAN: MIRA, Czech Republic). The samples were fixed on aluminium stubs with double adhesive tape and vacuum coated with a layer of gold before being visualized under a magnification of 2000×. Observations were carried out at acceleration voltages of 5 kV and 10 kV.

#### Stability of the encapsulated probiotics under gastro-intestinal (GI) tract conditions

This study investigated the survivability of the developed encapsulated probiotics in the GI tract as a continuous system. The method was developed using previously reported protocols^22, 23^. First, the dry powder of encapsulated probiotics (3 g) was mixed with 20 mL of saline solution (140 mmol/L KCl and 5 mmol/L NaCl) and agitated (95 rpm) at 37 °C for 10 min. The mixture was adjusted to pH 2 before 1 mL of gastric fluid (0.04 g/mL pepsin in 0.1 M HCl, pH 2.0) was added, and then the mixture was incubated under the previously mentioned conditions for 1 h. To simulate duodenal digestion, the mixture was then adjusted to pH 5.3 using 0.9 M sodium bicarbonate, 200 µL of bile salt (3 mg/L) and 100 µL of porcine pancreatin (80 mg/mL) were continuously added. The pH was adjusted to 7.4 with 1 M NaOH, and the mixture was continuously agitated for another 2 h. A sample (1 mL) was taken after incubation at the stages of gastric and intestinal digestion to determine the number of living probiotics. The survivability (%) of the probiotics after GI tract digestion was calculated using the following equation:

$${\text{Survivability }}\left( {\text{\% }} \right){ } = \left( {\frac{NP}{{IP}}} \right) \times 100$$

where NP is the number of probiotics recovered from each stage of digestion (gastric or intestinal) and IP is the initial number of probiotics. The survivability of the free cell probiotics under GI tract conditions was also determined as the control to study the effect of the encapsulation formulation. A total of three replicates of this experiment were performed.

#### Stability of the encapsulated probiotics during storage

To study the stability of the developed encapsulated probiotics, aluminium-laminated foil was selected as the effective packaging material to protect the encapsulated probiotics from the storage environment^24^. This evaluation was conducted at ambient temperature (28 ± 2 °C) and refrigeration temperature (6 ± 2 °C) over 8 weeks. The method by which the survivability of the encapsulated probiotics was determined was performed as mentioned in probiotic survivability assay after freeze-drying.

### Statistical analysis

Significant differences among the means of the results of short-chain fatty acid production by the tested bacterial strains at different times (1, 3, and 5 days) and the stability of various formulations of probiotics in the GI tract were calculated using Duncan’s multiple range tests at a 95% confidence level by IBM SPSS Statistics 28. The encapsulation of probiotics experiments were run in triplicate using simplex lattice mixture design (SLMD)^21^ to evaluate the effect of the ratio of wall material component (WPI, SO, and JS) on the probiotic recovery in dry powder form. EE (%) was considered as a response in this design.

## Results

### Selection and preparation of a probiotic cocktail

#### Ability of the probiotic strains to produce short-chain fatty acids (SCFAs)

**Figure 1:**
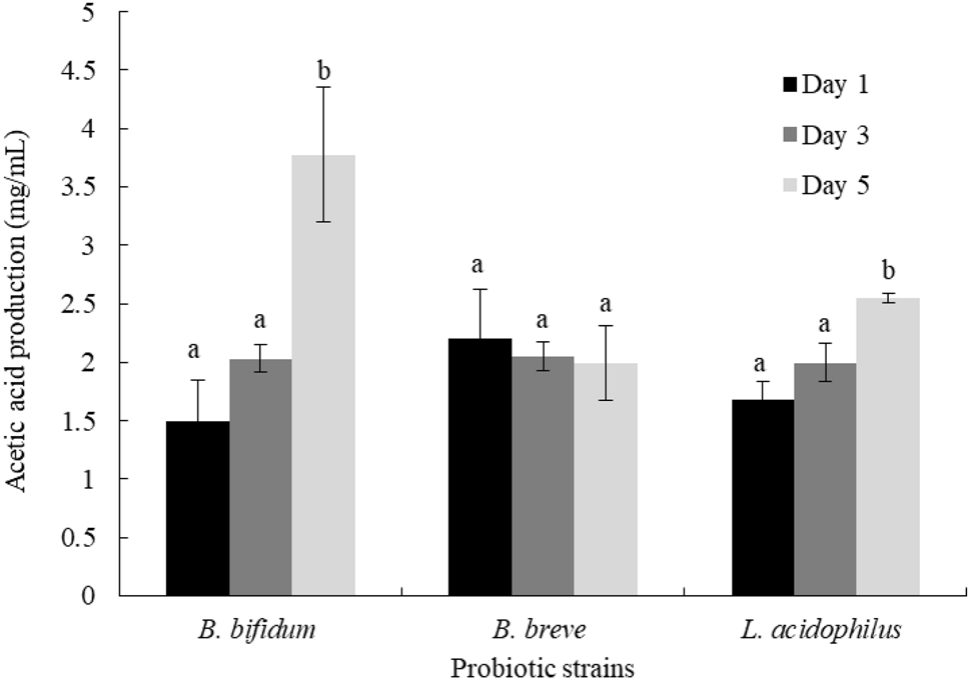
Acetic acid production of *B. bifidum*, *B. breve*, and *L. acidophilus* at days 1, 3, and 5 of incubation at 37 °C. Bars represent the mean standard deviation. Different lowercase letters indicate significant differences (P < 0.05) among incubation periods of each probiotic strain.

Acetic acid, butyric acid, and propionic acid have been reported to be beneficial SCFAs produced by beneficial bacteria in the gut. These SCFAs play an important role in several processes, especially lipid metabolism, resulting in human weight loss^25^. From our preliminary study on the production of acetic acid, butyric acid, and propionic acid, acetic acid was the only short-chain fatty acid detected, regardless of the strain used and incubation time (data not shown). The production of acetic acid from *B. bifidum*, *B. breve*, and *L. acidophilus* after incubation for 1, 3, and 5 days at 37 °C was then evaluated as shown in Fig. 1. After 5 days of incubation, *B. bifidum* and *L. acidophilus* showed significant increase of acetic acid production (P < 0.05), while there was no difference, comparing between those observed from day 1 and day 3 (P > 0.05). On the other hand, the similar acetic acid production was found from *B. breve* for every incubation time tested (P > 0.05).

#### Antibiotic-resistant profiles of the probiotic strains

Antibiotic resistance has become a major human health issue. Although using probiotics is a natural approach to improve human health, these beneficial bacteria have been reported to be reservoirs of antibiotic resistance determinants, especially in dietary supplements that contain multiple strains of probiotics^26^. The aim of this study was to develop an encapsulated probiotic formulation to improve dietary supplements in a further study. Thus, information on the antibiotic-resistant profiles of the tested probiotic strains is needed. In this study, a total of five antibiotics (ampicillin, gentamicin, erythromycin, streptomycin, and tetracycline) were used to investigate the antibiotic-resistant profiles of *B*. *bifidum*, *B*. *breve* and *L*. *acidophilus*. The antibiotic susceptibility assay conducted by the broth dilution method (Table 3) revealed that *B. bifidum*, *B. breve* and *L. acidophilus* were resistant to ampicillin (32 µg/L), streptomycin (512 µg/L) and gentamicin (32 µg/L), respectively.

**Table 3:** Antibiotic susceptibility of *B. bifidum*, *B. breve*, and *L. acidophilus*.

Bacterial strain	Antibiotic	Cutoff value (mg/L)^a^	MIC (mg/L)
*B. bifidum*	Ampicillin	2	32^b^
	Gentamicin	64	32
	Erythromycin	1	0.25
	Streptomycin	128	32
	Tetracycline	8	0.5
*B. breve*	Ampicillin	2	1
	Gentamicin	64	8
	Erythromycin	1	1
	Streptomycin	128	512^b^
	Tetracycline	8	4
*L. acidophilus*	Ampicillin	2	1
	Gentamicin	16	32^b^
	Erythromycin	1	1
	Streptomycin	16	16
	Tetracycline	4	4

^a^Cutoff value according to the European Food Safety Authority, 2018.

^b^Antibiotic-resistant strain.

#### Relationships among the probiotic strains

Combining effective bacterial strains has been recognized as a potential approach for a specific purpose. However, antagonism among bacterial cocktails needs to be identified. Bacteria can produce secondary metabolites that can act as inhibitory antibiotics or growth factors to support the growth of other bacteria in the same environment^27^. In this study, an aim was the development of multiple probiotic strains as a cocktail. Thus, the negative relationships between selected probiotics were determined. Antagonism among the tested probiotic strains was determined by the disk inhibition method (Table 4). The results showed an inhibition zone (approximately 0.2 mm) around the disks consisting of *B. breve* and *L. acidophilus* on *B. bifidum* lawns. This suggested that *B. breve* and *L. acidophilus* might produce metabolites that could inhibit the growth of *B. bifidum*. However, there were no inhibition zones around the *B.* *bifidum* disks on *B. breve* and *L. acidophilus* lawns. In addition, there was no evidence to indicate that *B. breve* and *L. acidophilus* inhibit each other. These results showed that *B. bifidum* should be excluded from the probiotic cocktail if it is to be used as the core material to be encapsulated.

**Table 4:** Antagonistic relationship among *B. bifidum*, *B. breve*, and *L. acidophilus*.

Potentially sensitive strain	Producer of inhibitory substance	Presence of inhibition zone^a^
*B. bifidum*	*B. breve*	+
	*L. acidophilus*	+
*B. breve*	*B. bifidum*	−
	*L. acidophilus*	−
*L. acidophilus*	*B. bifidum*	−
	*B. breve*	−

^a^+, Indicates the presence of an inhibition zone and −, indicates the absence of an inhibition zone.

### Development of a wall material made of jackfruit inner skin fibre, whey protein isolate and soybean oil for probiotic encapsulation

**Figure 2:**
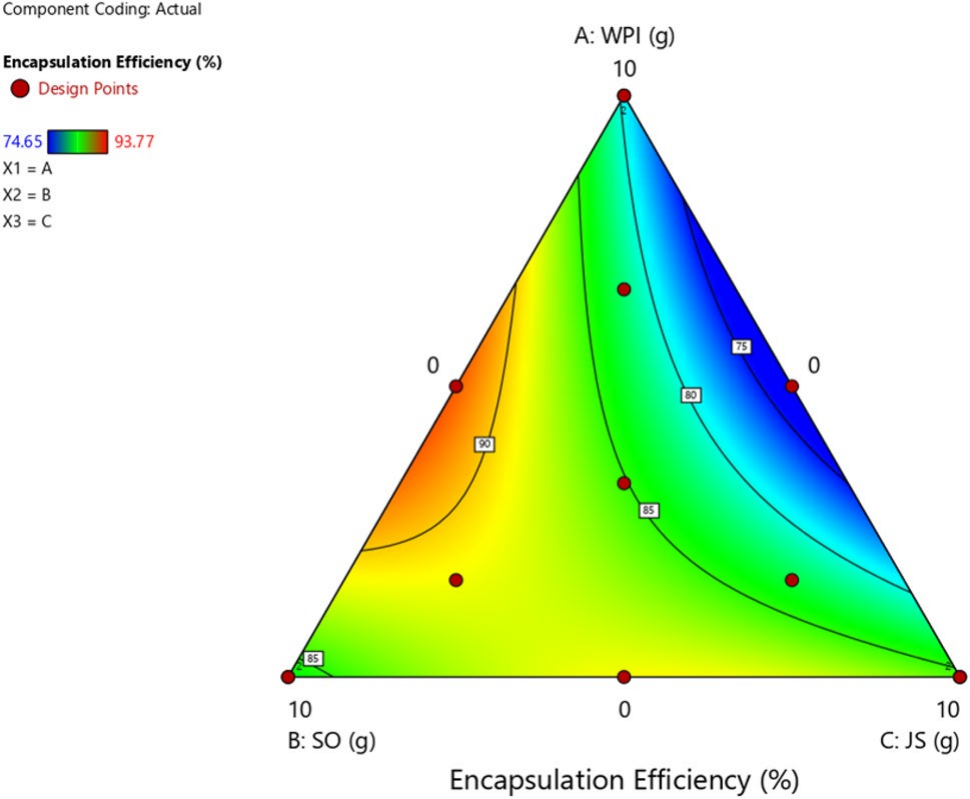
Mixture response surface contour plots displaying the combined effect of whey protein isolate (WPI), soybean oil (SO), and inner skin of jackfruit fibre (JS) on probiotic survivability recovered from dry powder after freeze-drying.

**Figure 3:**
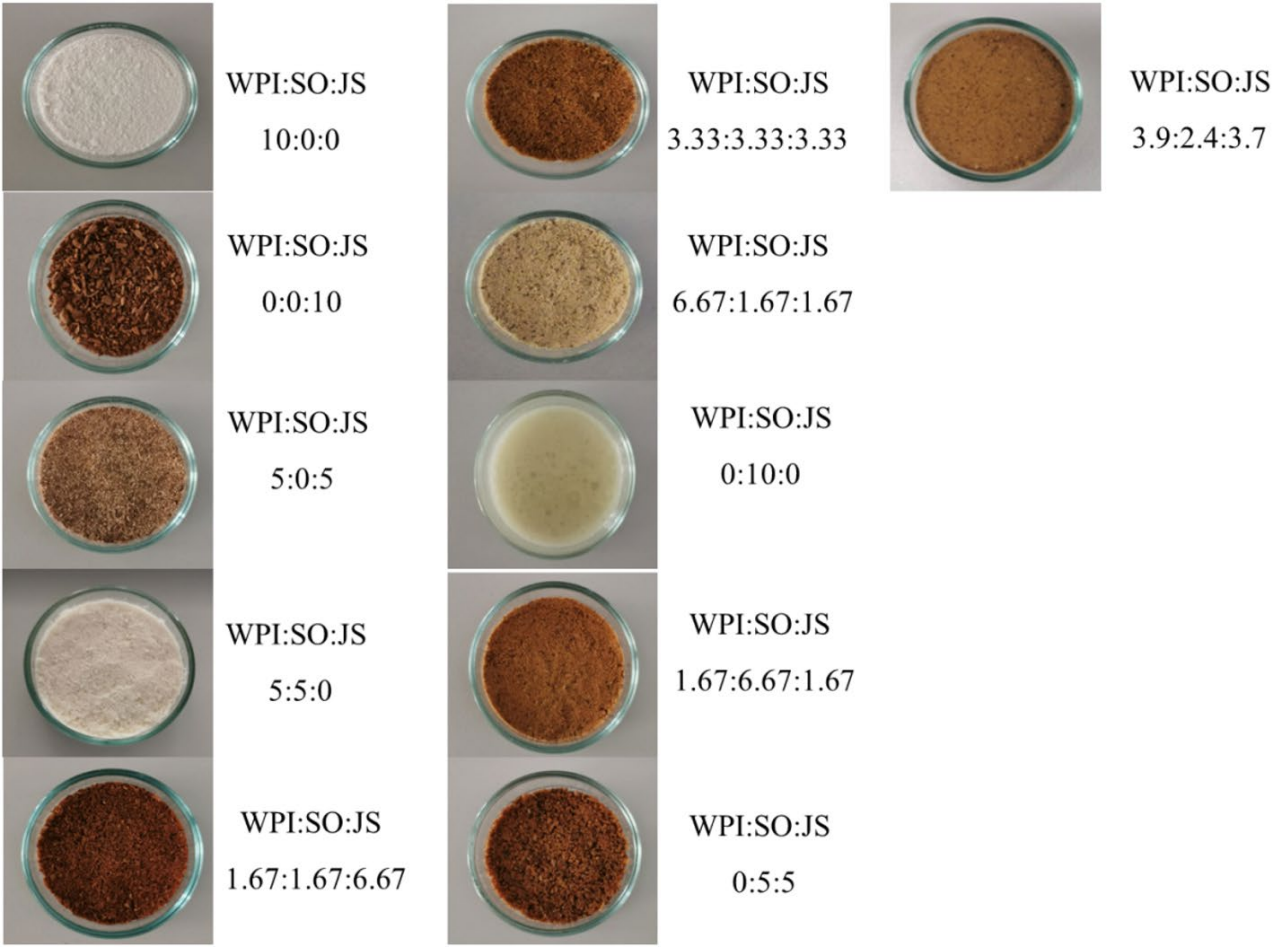
Characteristic of encapsulated probiotics with different wall materials formulated with various ratios of whey protein isolate (WPI), soybean oil (SO), and jackfruit inner skin fibre (JS).

Each wall material, WPI, SO and JS, was evaluated individually for its effect on the survivability of the probiotic cocktail (*B. breve* and *L. acidophilus*) under freeze-drying conditions. ANOVA revealed that the effect of the wall materials on probiotic survivability was as follows: % survivability =  + 7.9521WPI + 8.3847SO + 8.5732JS + 0.4171WPI*SO − 0.3894WPI*JS + 0.1637SO*JS (data not shown). This equation indicated that JS had the greatest positive effect on the survivability of the probiotics after freeze-drying, followed by SO and WPI. In addition, combinations of these 3 different wall materials were also studied, and the efficiencies of 13 formulations with 10 different ratios of WPI, SO, and JS were evaluated (Table 2). The encapsulation efficiency (EE %) ranged from 74.65 ± 3.97 to 93.77 ± 4.06 (Fig. 2). Moreover, the results showed that the combinations WPI-SO and SO-JS also produced positive effects. However, the combination of WPI and JS showed a negative effect that decreased the likelihood of the probiotics surviving under freeze-drying conditions. Although WPI-SO at an equal ratio showed the highest EE among the 10 tested formulations (93.77 ± 4.06%; data not shown), in this study, it was noted that JS was the source of prebiotics and SO and WPI maintained the encapsulation efficiency and transformed the encapsulated probiotics into dry powder (Fig. 3). Thus, the optimal formulation should contain all of the tested wall materials. The optimal formulation of the encapsulated probiotics was designed based on its limitations. Thus, a formula with WPI, SO, and JS ratios of 3.9, 2.4, and 3.7, respectively, was designed. This formulation was then developed and showed an encapsulation efficiency of 83.1 ± 6.1%. The optimal formulation was further characterized in terms of its physical morphology to investigate its efficiency to protect probiotics during GI tract digestion. Finally the stability of encapsulated probiotics during storage was evaluated under different temperatures.

### Characterization and stability studies of the encapsulated probiotics

#### Morphology and surface characterization of the encapsulated probiotics

**Figure 4:**
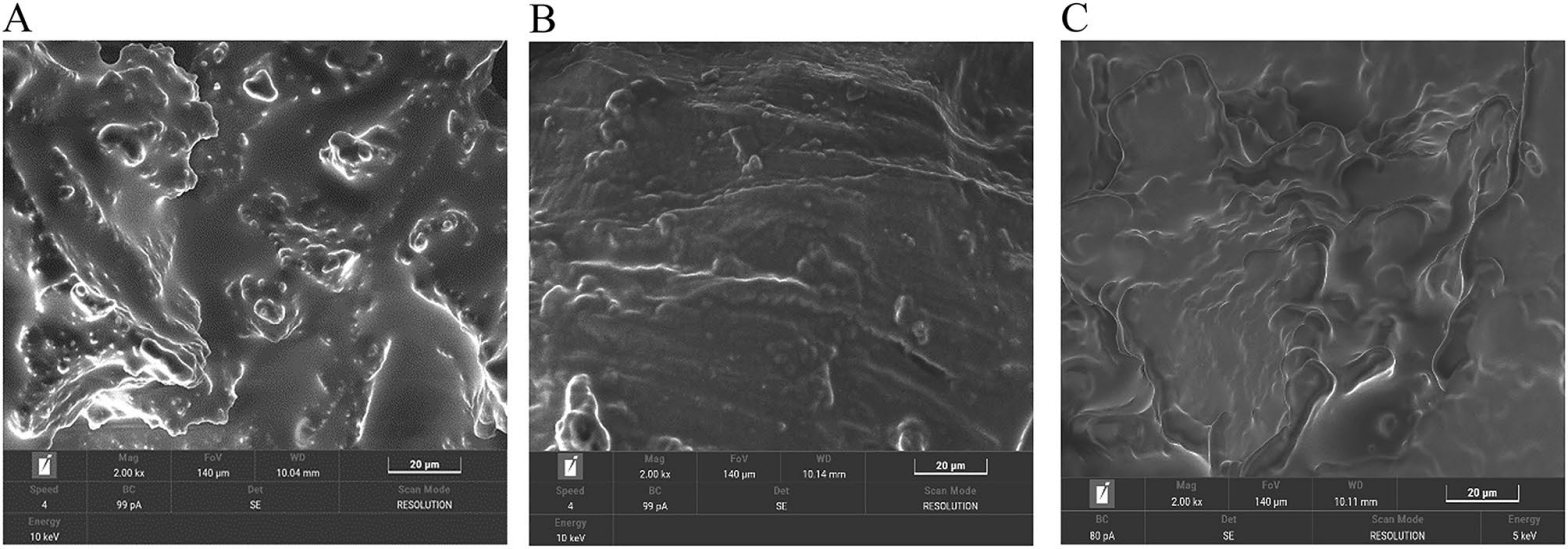
Scanning electron microscopic photographs at 2000× magnification of encapsulated probiotics. WPI-SO (**A**); encapsulated probiotics with whey protein isolate and soybean oil at 1:1 ratio, JS-SO (**B**); encapsulated probiotics with jackfruit inner skin fibre and soybean oil at 1:1 ratio, Optimal formulation (**C**); encapsulated probiotics with whey protein isolate, soybean oil, and jackfruit inner skin fibre at 3.9:2.4:3.7 ratio.

Morphological images of the probiotics encapsulated in the optimal formulation compared to probiotics encapsulated with the WPI-SO and JS-SO formulations are shown in Fig. 4. The probiotics encapsulated by the WPI-SO formulation showed a rough surface with pores of various sizes in the structure. The JS-SO formulation also showed a rough surface as well as the presence of cracks and a fibrous structure. Interestingly, the optimal formulation showed a uniform morphology with a smooth and dense matrix.

#### Stability of the encapsulated probiotics under stimulated GI tract conditions

**Figure 5:**
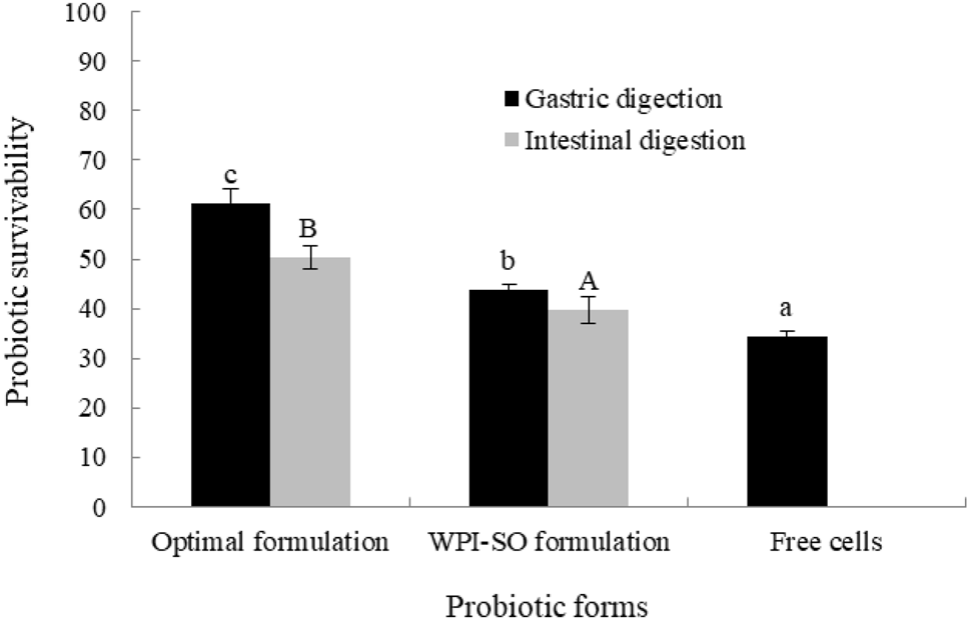
Survivability of encapsulated probiotics with optimal formulation under stimulated continuous gastro-intestinal tract in-vitro compared to WPI-SO formulation, and free cells. Bars represent the mean standard deviation. ^a,b,c^ indicated significant differences (P < 0.05) between the sample in gastric digestion condition. ^A,B,C^ indicated significant differences (P < 0.05) between the sample in intestinal digestion condition.

This study simulated the conditions of GI tract digestion as a continuous system. Probiotics encapsulated by the optimal formulation and the WPI-SO formulation and free-cell probiotics were studied to demonstrate the effect of JS as a potential wall material to protect the probiotics in the GI tract. The optimal formulation provided the probiotics with remarkable protection from harsh conditions (Fig. 5). After incubation in gastric fluid (pH 2) for 1 h, the reduction in probiotics released from the optimal formulation was 3.2 ± 0.4 log CFU/mL (62.3 ± 2.7% survivability). After intestinal digestion, the total reduction probiotics encapsulated by the optimal formulation lead to 4.2 ± 0.4 log CFU/mL (50.4 ± 2.3% survivability). The probiotics encapsulated by the WPI-SO formulation were studied in this assay because this formulation had the highest %EE. The reduction in probiotics after exposure to gastric fluid and intestinal digestion was 5.3 ± 0.2 log CFU/mL (43.8 ± 1.1% survivability) and 5.7 ± 0.3 log CFU/mL (39.1 ± 2.7% survivability), respectively. The survivability of free-cell probiotics under GI tract conditions showed a reduction in 7.2 ± 0.1 log CFU/mL (34.5 ± 1.1% survivability) after treatment with gastric fluid. Moreover, unencapsulated probiotics could not survive after intestinal tract digestion.

#### Stability of the encapsulated probiotics during storage

**Figure 6:**
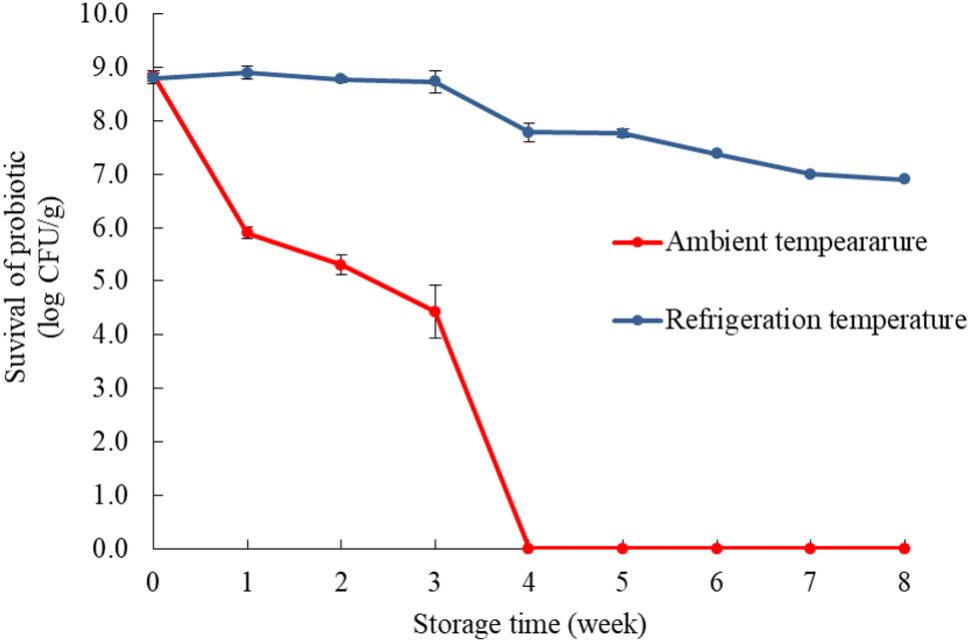
Stability of encapsulated probiotics kept in aluminium-laminated foil bags at ambient temperature (28 ± 2 °C) and refrigeration temperature (6 ± 2 °C) for 8 weeks.

This study used aluminium-laminated foil bags as the packaging material for the developed encapsulated probiotics due to reports that suggest that this form of packaging can effectively protect encapsulated microorganisms from moisture, oxygen, and light^24^. Furthermore, this study evaluated the stability of the encapsulated probiotics at ambient and refrigeration temperatures for 8 weeks (Fig. 6). At an ambient temperature, an approximate 3 log reduction was observed after 1 week of storage, compared with that of 0 week of storage. Moreover, surviving probiotics were not detected after 4 weeks of storage. Remarkably, storage at refrigeration temperature showed a reduction in probiotic survival by approximately 2 log reduction (77.8 ± 0.1% of survivability) after 8 weeks. Thus, it is recommended that such developed products be stored at a low temperature to extend probiotic viability.

## Discussion

The encapsulated probiotics in this study showed desirable properties that can be improved for use as food supplements. The production of acetic acid in this study was higher than that found in other studies. The amount of acetic acid produced by *B.* *bifidum* ATCC 29521 cultured with fructooligosaccharide at 37 °C for 48 h was 3.17 mg/mL^28^, and after *L. acidophilus* ATCC 11975 was cultured in MRS broth at 37 °C for 24 h, acetic acid production was 0.82 mg/mL^29^. Acetic acid can inhibit the growth of intestinal pathogens, provide energy to the liver and surrounding tissues, and it play a vital role in gluconeogenesis and lipogenesis^30, 31^. Therefore, this important short-chain fatty acid could play an important role in the reduction of metabolic regulation in humans. Information related to the antibiotic-resistant profiles of individual probiotic materials has remained scarce^32^. This study performed an in-vitro screening of the antibiotic-resistant profiles of certain bacteria to provide beneficial data on probiotic characteristic-related issues of concern. Although the probiotic strains in this study showed resistance to a few antibiotics, a previous study reported that probiotics that harbour resistance genes are themselves not harmful^33^. In addition, most probiotics, especially lactic acid bacteria (LAB), which were used in this study, have been considered to have generally recognized as safe (GRAS) status, which allows them to be used in food products^33, 34^. The results of this study agree with those a previous study that reported the importance of encapsulation to enhance the viability of probiotics under harsh conditions^35^. The probiotics used in this study were gram-positive bacteria that contained a thick peptidoglycan layer on the cell wall. The peptidoglycan layer comprises linear polysaccharide chains cross-linked with small peptides, which are covalently linked to teichoic acids^36^. This cross-linked network formed by H-bonding between the hydroxyl groups of polysaccharides (contained in the jackfruit inner skin fibre) might provide protection by entrapping probiotic cells in the porous structure of the polysaccharides. The cell walls of gram-positive bacteria contain small portions of proteins and lipids. An oil phase can interact with the hydrophobic portions of proteins and the nonpolar structures of the lipids on the bacterial cells. WPI has been reported to be an effective wall material that can protect microorganisms from potential protein‒protein interactions^24^. The results here indicated that WPI possibly covered the probiotic cells by interacting with the short peptides on the bacterial cell wall, thus protecting the probiotic cells from dry conditions. The protein molecules could have interacted with the polysaccharide as a gel complex, resulting in a decrease in the size of the pores in the network^37^. This phenomenon might be a barrier to probiotic cell entrapment, leading to probiotic cells being directly exposed to harsh conditions. Therefore, the protein structure may act as a buffer to protect living cells from the extreme pH of the gastric fluid^38^. The combination of a protein with the polysaccharide may have affected the release rate of the probiotics during simulated gastrointestinal digestion, leading to the strong protection of the encapsulated probiotics^10^. Encapsulated probiotics as hydrogel which contained protein based-materials (whey protein isolate and soy protein isolate) have been reported to be effective wall materials to protect probiotics from the GI tract^39, 40^. In addition, the synergistic effect of the combination of the protein, oil, and polysaccharide efficiently protected the probiotics from the drying process and GI tract^10, 13^. The storage stability evaluation results showed that the encapsulated probiotics in this study met the regulations for use in food, which state that foods containing probiotics must have no less than 10^6^ CFU/g live probiotics throughout the shelf life of the product^41^. Overall, jackfruit inner skin fibre could be an effective wall material for probiotic encapsulation, particularly if it is incorporated with other commercial materials, such as whey protein isolate and soybean oil. Encapsulation formulation and process developed in this study was simple and provided high probability to scale-up. Moreover, the current formulation and process to develop encapsulated probiotics is of interest to improve as functional food for weigh control.

## Conclusion

Jackfruit inner skin fibre (JS) successfully improved the efficiency of whey protein isolate and soybean oil as wall materials to protect probiotics from freeze-drying and GI tract conditions. The optimal formulation with WPI:SO:JS at 3.9: 2.4: 3.7 ratio showed high efficiency in protecting probiotics from freeze-drying conditions (83.1% survivability), gastric- and intestinal- digestion (62% and 50% survivability, respectively) as well as refrigeration storage (77.8% survivability). Therefore, JS could be a useful prebiotic source with a good protective effect as well as a wall material for the encapsulation of probiotics, particularly incorporation with other commercial materials as WPI and SO.

## Data Availability

The datasets generated in the current study are available from the corresponding author on reasonable request.
